# Experimental Study on the Effect of Urban Road Traffic Noise on Heart Rate Variability of Noise-Sensitive People

**DOI:** 10.3389/fpsyg.2021.749224

**Published:** 2022-01-11

**Authors:** Chao Cai, Yanan Xu, Yan Wang, Qikun Wang, Lu Liu

**Affiliations:** School of Architecture, Tianjin Chengjian University, Tianjin, China

**Keywords:** road traffic noise, noise sensitivity, indoor-level noise, heart rate variability, acute physiological effect

## Abstract

Epidemiological studies have confirmed that long-term exposure to road traffic noise can cause cardiovascular diseases (CDs), and when noise exposure reaches a certain level, the risk of related CDs significantly increases. Currently, a large number of Chinese residents are exposed to high noise exposure, which could greatly increase the risk of cardiovascular disease. On the other hand, relevant studies have found that people with high noise sensitivity are more susceptible to noise. And it is necessary to pay more attention to the high noise-sensitive people. This study investigated the acute physiological effect of different noise-sensitive groups by indoor-level noise stimulus experiments under laboratory conditions, by observing heart rate variability (HRV) indicators, including standard deviation of NN intervals (SDNN), low frequency/high frequency (LF/HF), and heart rate (HR). The results showed that (a) there was no significant difference in HRV between the high-sensitive group and the low-sensitive group at the physiological baseline and the different stimulating noise levels. (b) Then, based on the theory of cumulative effect of noise proposed by WHO Regional Office for Europe, non-significant but observable differences between groups were further discussed. By analyzing differences of the variation trends and the within-group significant changes of SDNN and HR between the two groups, the results tended to show that the high-sensitive group is more affected by road traffic noise. In addition, the values of SDNN and HR showed observable between-group differences at 55 dB (A) and 65 dB (A) which corresponding to the SPL associated with a significantly increased risk of cardiovascular disease concerned by epidemiological studies. According to the cumulative effect theory (WHO), these differences in HRV caused by short-term noise stimulation may have the potential to produce physiological response and lead to between-groups differences in prevalence after long-term recurrent effect, and deserve attention and further research.

## Introduction

Road traffic noise is one of the causative factors for cardiovascular diseases (CDs) confirmed by numerous studies (Babisch, 2011; Basner et al., 2014). According to the previous research conclusions of noise exposure limits, a considerable number of residents in Chinese cities are exposed to high risk noise environment, facing the greater risks of CDs (Hu et al., 2019). Meanwhile, because high-noise sensitive people are more susceptible to noise, showing a higher level of psychological and physiological influence than others (Persson et al., 2007), the physiological effects of noise related to CDs in this population should be concerned and studied.

Nowadays, the exposure of urban road traffic noise in China is relatively severe (Yang, 2020), which has reached or exceeded the noise control recommendations proposed by relevant studies. According to the China Environmental Noise Prevention and Control Annual Report of the Ministry of Ecology and Environment of the People’s Republic of China (2019), from 2015 to 2019, the *L*_d_^1^ of road traffic noise in cities nationwide was 66.8 dB (A) to 67.1 dB (A), and the *L*_d_ in first-tier cities was higher, reaching at 68.5 dB (A) to 68.9 dB (A). For another, previous epidemiological studies have preliminarily confirmed that long-term exposure to road traffic noise mainly causes CDs (Sørensen et al., 2013; Basner and McGuire, 2018), especially the daytime noise exposure (WHO Regional Office for Europe, 2018b). And some conclusions have shown that the risk of CDs significantly increases when *L*_den_ is higher than 55 dB (A) or 60 dB (A) (Bluhm and Eriksson, 2011). For the sake of protecting human health, World Health Organization (WHO) proposed the noise limit (*L*_den_) of 53 dB (A) in 2018. However, due to the lack of relevant researches in China, especially in the face of the poor road traffic noise environment, more attention should be paid to the impact of road traffic noise on Chinese people.

Different noise-sensitive people are affected differently by noise. As an independent personality trait and a potential variable of individual, noise sensitivity plays a key role in studies on noise annoyance and noise-induced health deterioration (Smith, 2003), and has been gradually paid attention to in studies of the noise impact on the public as an observing factor (van Kamp and Davies, 2013). Previous epidemiological and physiology-psychology studies have found that compared with low-noise sensitive people, high-noise sensitive people have higher subjective annoyance (Jong and Jin, 2011), higher perceived stress (Fyhri and Aasvang, 2010), while worse sleep quality (Halonen et al., 2012) and lower cognitive level (Wright et al., 2014). And physiologically, high-noise sensitive people are at higher risk of CDs (Babisch et al., 2009; Berry and Flindell, 2009; Ndrepepa and Twardella, 2011). These studies indicate that physiological effects of noise are related to noise sensitivity, and more attention should be paid to high-noise sensitive people.

Besides, the relevant research methods of acute effect is available to explore the physiological effects of noise, which is conducive to the observation of indicator variation trend and sensitivity differences in short period (Buccelletti et al., 2009; Lee et al., 2010). For example, numerous researchers have found physiological trends in CDs [through heart rate variability (HRV) indicators] with noise exposure in short-term strong noise stimulation studies (Lusk et al., 2004; Björ et al., 2007; Haralabidis et al., 2008; Dirk et al., 2010). Meanwhile, HRV is often used as a physiological indicator reflecting cardiovascular disease in the noise evaluation of physiological effects, which is an effective indicator to judge and predict cardiovascular diseases in medicine and is reliable physiological parameters to measure the physiological stress state of the human body under noise environment (Lan, 2010). Overall, HRV, as a physiological indicator suitable for observing short-term noise stimulation, is available for this study.

Thus this study focused on the cardiovascular effects of road traffic noise induced in noise-sensitive people through short-term noise stimulation experiment in laboratory, and compared and discussed the physiological effect of noise between high-sensitive groups and low-sensitive groups.

## Materials and Methods

The noise stimuli experiments were carried out in a semi-anechoic laboratory, with the HRV index measures as the experimental physiological indicators. According to WHO Regional Office for Europe (2018a), chronic CDs are primarily associated with sustained daytime noise exposure, this study explored the influence of noise on subjects in the daytime awake state. The reading state was taken as the test state, and neutral and calm current events were selected as reading materials, in order to minimize the influence of non-experimental factors on the study. A repeated-measures ANOVA was used to analyze the data of the indicators to determine the physiological effect of road traffic noise on people with different noise sensitivities.

### Experimental Factor and Levels

The experimental factor was the sound pressure level (SPL), using *L*_Aeq_ as the corresponding evaluating indicator. Due to the majority of road traffic noise complaints occurred inside buildings according to the China Environmental Noise Prevention and Control Annual Report, this study focused on the physiological effect of noise under the indoor environment. The exposure SPL levels in this study were set to match the following *L*_Aeq_ values: 35 dB (A), 45 dB (A), 55 dB (A), 65 dB (A), based on the field measured data of indoor SPL [*L*_d,max_ = 62.4 dB (A) and *L*_d,min_ = 33.7 dB (A)] and the laboratory background SPL [15.7 dB (A), set as the physiological resting state SPL level]. The experimental noise signals were live audio recordings, and played by Adobe Audition CC2019, JTS-PA150 audio power amplifier, JTSY-omnidirectional sound source which is passive speaker (Beijing J.T Technology Co., Ltd.), and the audio interface is bayonet nut connector (BNC).

Noise signal recording and indoor SPL testing were performed in a typical frontage residential unit with the window opened. The test site is located at an urban expressway in Tianjin, China, which have eight-lane in both directions, with an average daily traffic flow of 86,940 vehicles, including 15,395 heavy vehicles. The noise signals were recorded by Roland R44 with Rode NTG-3 microphone (1 channel, 24 bit rate, 96 kHz sampling frequency), then through the distortion degree analysis, the signals with distortion degree less than 3% were selected (Xie, 2006). After modulating the SPL by spectraLAB, the final experimental noise signals were made (89.14% low-frequency energy, non-stationary noise). Due to the focus on the physiological effects of SPL factors, noise spectrum and other characteristics were not considered in this study.

### Experimental Observation Indicators

Heart rate variability, as a reliable physiological parameters to measure the physiological stress state of human body under noise environment, were selected as the experimental observation indicators in this study, included time domain parameter [Standard Deviation of NN intervals (SDNN)], frequency domain parameter [Low Frequency/High Frequency (LF/HF)], and Heart Rate (HR). Among them, the SDNN is stable and has a good correlation in the repeat evaluation (HRV Co-operation Study Group, 2000). LF/HF is a valid evaluation index of increased sympathetic activity and decreased parasympathetic activity, and has been widely used in related studies.

This research adopted the ErgoLAB Man-Machine-Environment Testing Cloud Platform V3.0 (Kingfar International Inc., Beijing, China) to record the physiological indicators. And that includes the design module of ErgoLAB V3.0, ECG sensors from the wearable physiological recording system, and analysis modules for HRV, which is used to process data.

### Experimental Subjects and Noise Sensitivity Grouping

College students were selected for this study who are convenient for experimental observation as the experimental subjects, because they are relatively healthy and have no cardiovascular diseases compared with other age groups in the adult population. A total of 30 college students were randomly selected as the experimental subjects (mean age of 23.1 ± 1.41 years; 17 male and 13 female). No subjects had past medical history of CDs and all subjects had normal basic hearing. Before the experiment, noise sensitivity of all subjects were collected by the Weinstein Noise Sensitivity scale (Zhong et al., 2018; Moghadam et al., 2021), noise sensitivity grouping was performed according to the scale score. And cluster analysis revealed a noise sensitivity grouping value of 69 points (*p* = 0.000). Fifteen subjects with scores ≥69 were classified as the high-sensitivity group, and fifteen subjects with scores ≤68 were classified as the low-sensitivity group.

### Experimental Process Control

To reduce the experimental error as much as possible, the following measures were taken to control the experimental procedure.

#### Experimental Steps and Duration

**FIGURE 1:**
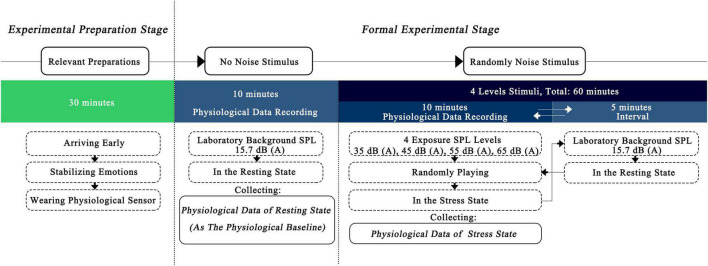
Flow chart of experimental steps.

The main steps of the formal experiment were as follows (Figure 1):

(1) On the day of the experiment, participants arrived early at the laboratory to make relevant preparations before the experiment, including wearing physiological sensor, gradually adapting to the environment, and stabilizing their emotions. This process lasted for 30 min.
(2) Without playing any experimental noise signal, the physiological data of the subjects in the resting state were recorded for 10 min.
(3) In the formal noise exposure experiment, physiological data of the stress state were collected and recorded during exposure to experimental noise signals of different SPL levels that were randomly played one by one. The noise exposure lasted for 10 min, and the interval between two stimuli was 5 min. The relevant time length was set according to the following bases:

• The basis for setting the duration of a single noise exposure.

A report published by the WHO Regional Office for Europe (WHO Regional Office for Europe, 2018a) showed that acute physiological effects can occur within seconds or minutes from the initiation of a noise stimulus. In addition, in the relevant research of acute effect, lower SPL levels have been studied with noise exposure duration of mostly 10–20 min (Walker et al., 2016), while higher noise exposure duration was mostly 5–10 min (Kraus et al., 2013; El Aarbaoui et al., 2017). Therefore, the duration of a single noise exposure was set to 10 min.

• The basis for setting the duration of the interval between two noise stimuli.

The pre-experiment found that the HRV indicators, gradually stabilized between 3 and 5 min after exposure to noise. Therefore, the interval between two noise stimuli was set to 5 min.

#### Experimental Environment Control

**FIGURE 2:**
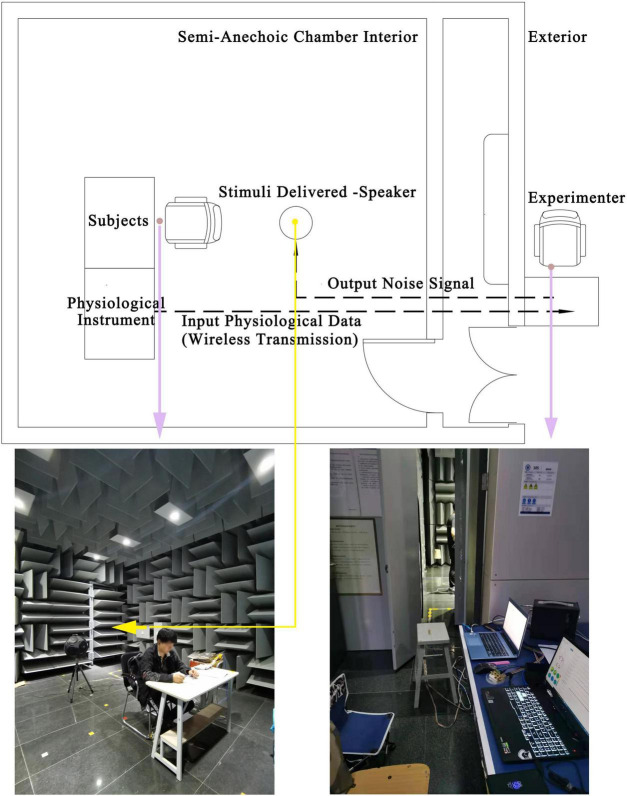
Schematic diagram of the experimental environment control.

The experimental environment control included the following three main aspects:

(1) The noise exposure experiments were completed in a semi-anechoic laboratory, which can meet the demand for playback of low SPL level noise signals.
(2) The light and thermal environment of the laboratory were strictly controlled to ensure that the relevant environmental parameters were maintained at comfort psychological and physiological levels. The horizontal illumination of the desktop on which to complete reading tasks was set at 735 Lux, the air temperature was around 25°C, and the relative humidity of the air was 50–60%.
(3) The experiment-related equipment was placed outside the laboratory to avoid disturbance to the subjects by the sound of operating the equipment and experimenter activity. The physiological data were collected through wearable sensors and wirelessly transmitted to the recording (Figure 2).

#### Experimental Subject Control

The following measures were also taken to avoid unnecessary psychological fluctuations:

(1) One week before the formal experiment, subjects familiarized themselves with the laboratory environment, and explanations about the experimental process were provided.
(2) One day before the formal experiment, subjects were asked not to drink alcohol, tea, coffee, or other caffeinated beverages, and to ensure that they had sufficient sleep the night before the experiment.
(3) Half an hour before the formal experiment, subjects were asked not to perform strenuous exercise and to arrive at the laboratory in advance to prepare and adapt to the environment.

## Results

### The Differences in Significance of Heart Rate Variability Between Noise-Sensitive Groups

**FIGURE 3:**
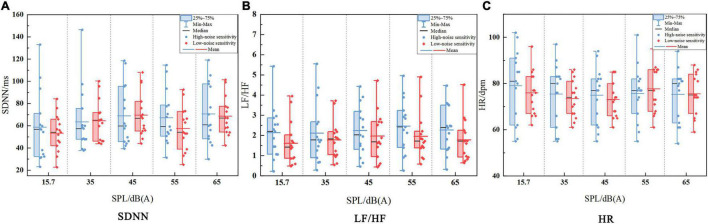
The distribution of HRV indicators of the subjects in different noise-sensitive groups. **(A)** The distribution of SDNN in different noise-sensitive groups. **(B)** The distribution of LF/HF in different noise-sensitive groups. **(C)** The distribution of HR in different noise-sensitive groups.

Figure 3 shows the distributions of HRV with the stimulating SPL changing in the high-sensitive group and the low-sensitive group, including the value of maximum, minimum, median, quartile, mean and other information. And the result of one-sample Kolmogorov–Smirnov test (Table 1) showed that the distribution of observed physiological data of all experimental samples met the normal distribution. Then the result of the further repeated-measures ANOVA (Table 2) showed that the *p*-values of all HRV indicators were greater than 0.05, indicating that there was no significant difference in HRV between the two noise-sensitive groups under short-term noise stimulation. This result has two implications: First, there were no significant differences in physiological baseline^2^ of the SDNN, LF/HF, and HR between two groups. Second, there were no significant differences in the SDNN, LF/HF, and HR responses to the same level of noise stimulus between two groups.

**TABLE 1:** One-sample Kolmogorov–Smirnov test results.

SDNN	15.7 dB	35 dB	45 dB	55 dB	65 dB
Exact sig. (two-tailed)	0.505	0.290	0.429	0.810	0.716
LF/HF	15.7 dB	35 dB	45 dB	55 dB	65 dB
Exact sig. (two-tailed)	0.693	0.312	0.852	0.791	0.842
HR	15.7 dB	35 dB	45 dB	55 dB	65 dB
Exact sig. (two-tailed)	0.916	0.713	0.933	0.984	0.519

**TABLE 2:** The repeated-measures ANOVA results.

	SDNN	LF/HF	HR
*F*	0.056	1.613	0.147
Sig.	0.814	0.214	0.705

*p > 0.05, no significant difference.*

The results of non-significant differences between groups were inconsistent with the conclusions of previous relevant studies. And the possible reasons for these results will be analyzed and discussed in the section “Non-significant Difference Between Noise-Sensitive Groups” of this manuscript.

### Variation Trend of Heart Rate Variability in the High-Sensitive Group

#### Standard Deviation of NN Intervals

**FIGURE 4:**
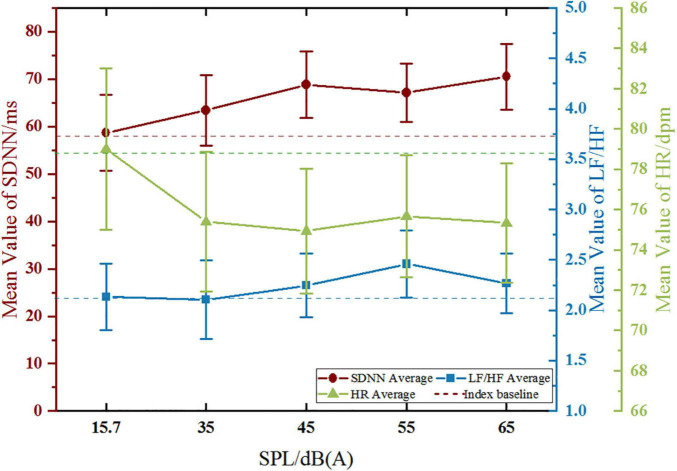
Variation trend of HRV with SPL in the high-sensitive group.

The variation trend of SDNN in the high-sensitive group with SPL is shown in Figure 4. With the increase of SPL, SDNN generally presented an upward trend. The SDNN increased significantly from 15.7 dB (A) to 45 dB (A), and this upward trend eased off from 45 dB (A) to 65 dB (A), with slight fluctuations. The maximum value of SDNN offset to the baseline appeared at 65 dB (A). The repeated-measures ANOVA result of SDNN for the main effect of SPL was *p* = 0.043, and the corresponding results of pairwise comparison (Table 3) revealed that the SDNN only showed a significant difference between 45 dB (A) and 15.7 dB (A) (*p* < 0.05).

**TABLE 3:** High-sensitive group’s SDNN/ms pairwise comparisons.

(I) SPL	(J) SPL	Mean difference (I-J)	Std. error	Sig.^b^	95% confidence interval for the difference^b^	
					Lower-bound	Upper-bound
15.7 dB (Resting)	35 dB	–4.731	3.003	0.137	–11.172	1.709
	45 dB	−10.133*	3.547	0.013	–17.741	–2.526
	55 dB	–8.451	4.757	0.097	–18.654	1.752
	65 dB	–11.836	5.646	0.055	–23.945	0.273
35 dB	45 dB	–5.402	4.002	0.199	–13.986	3.182
	55 dB	–3.720	4.503	0.423	–13.377	5.937
	65 dB	–7.105	5.430	0.212	–18.751	4.542
45 dB	55 dB	1.682	4.408	0.709	–7.773	11.137
	65 dB	–1.703	5.484	0.761	–13.465	10.06
55 dB	65 dB	–3.385	3.661	0.371	–11.236	4.467

*Mauchly’s test of sphericity p = 0.257 > 0.05 obeyed the hypothesis of a spherical distribution, and the tests of within-subjects effects was p = 0.043 < 0.05. Based on estimated marginal means.*

*The mean difference was significant at the 0.05 level.

*^b^Adjustment for multiple comparisons: Least Significant Difference (equivalent to no adjustments).*

#### Low Frequency/High Frequency

The variation trend of LF/HF in the high-sensitive group (Figure 4) showed that LF/HF gentle increase at first and then decreased with the increase of SPL. The LF/HF increased obviously from 35 dB (A) to 55 dB (A), and the maximum value of LF/HF offset to the baseline occurred at 55 dB (A). The Mauchly’s test of sphericity of LF/HF (*p* = 0.010 < 0.05) did not obey the hypothesis of spherical distribution, and the further multivariate test was *p* = 0.939, which indicated that there was no significant difference in LF/HF between the different SPL levels.

#### Heart Rate

The variation trend of HR in the high-sensitive group (Figure 4) showed a general downward trend with the increase of SPL, whereby HR decreased significantly from 15.7 dB (A) to 35 dB (A), and this downward trend began to plateau from 35 dB (A) to 65 dB (A), with slight fluctuations. The maximum value of HR offset to the baseline appeared at 45 dB (A). The repeated-measures ANOVA result of HR for the main effect of SPL was *p* = 0.050, and the corresponding results of pairwise comparison (Table 4) revealed that HR showed significant differences between 35 dB (A), 45 dB (A), and 15.7 dB (A) (*p* < 0.05).

**TABLE 4:** High-sensitive group’s HR/bpm pairwise comparisons.

(I) SPL	(J) SPL	Mean difference (I-J)	Std. error	Sig.^b^	95% confidence interval for the difference^b^	
					Lower-bound	Upper-bound
15.7 dB (Resting)	35 dB	3.600*	1.129	0.007	1.179	6.021
	45 dB	4.067*	1.152	0.003	1.595	6.539
	55 dB	3.333	2.670	0.232	−2.394	9.060
	65 dB	3.667	2.203	0.118	−1.059	8.392
35 dB	45 dB	0.467	0.888	0.608	−1.439	2.372
	55 dB	–0.267	2.159	0.903	−4.897	4.364
	65 dB	0.067	1.551	0.966	−3.259	3.392
45 dB	55 dB	–0.733	2.161	0.739	−5.369	3.902
	65 dB	–0.400	1.650	0.812	−3.939	3.139
55 dB	65 dB	0.333	1.027	0.750	−1.869	2.535

*Mauchly’s test of sphericity p = 0.000 < 0.05 did not obey the hypothesis of spherical distribution, and the multivariate test results was p = 0.050. Based on estimated marginal means.*

*The mean difference was significant at the 0.05 level.

*^b^Adjustment for multiple comparisons: Least Significant Difference (equivalent to no adjustments).*

### Variation Trend of Heart Rate Variability in the Low-Sensitive Group

#### Standard Deviation of NN Intervals

**FIGURE 5:**
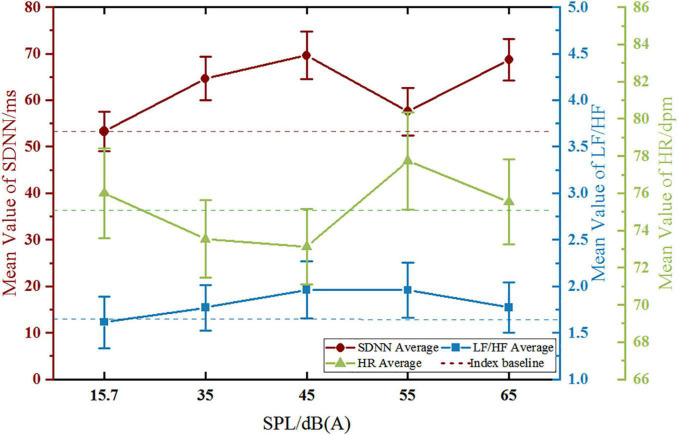
Variation trend of HRV with SPL in the low-sensitive group.

The variation trend of the SDNN in the low-sensitive group with SPL is shown in Figure 5. With the increase of SPL, the SDNN generally showed an upward trend. The SDNN increased significantly from 15.7 dB (A) to 45 dB (A), and this upward trend eased off from 45 dB (A) to 65 dB (A), with a more obvious decline at 55 dB (A). The maximum value of the SDNN offset to the baseline appeared at 45 dB (A). The repeated-measures ANOVA result of SDNN for the main effect of SPL was *p* = 0.004, and the corresponding results of pairwise comparison (Table 5) revealed that significant differences in the SDNN were found between 35 dB (A), 45 dB (A), 65 dB (A), and 15.7 dB (A) (*p* < 0.01), as well as between 45 dB (A), 65 dB (A), and 35 dB (A) (*p* < 0.05), between 55 dB (A) and 45 dB (A) (*p* < 0.05), and between 65 dB (A) and 55 dB (A) (*p* < 0.05).

**TABLE 5:** Low-sensitive group’s SDNN/ms pairwise comparisons.

(I) SPL	(J) SPL	Mean difference (I-J)	Std. error	Sig.^b^	95% confidence interval for the difference^b^	
					Lower-bound	Upper-bound
15.7 dB (Resting)	35 dB	−11.341*	3.120	0.003	–18.033	–4.650
	45 dB	−16.343*	3.352	0.000	–23.533	–9.154
	55 dB	–4.248	3.659	0.265	–12.095	3.599
	65 dB	−15.360*	3.424	0.001	–22.704	–8.016
35 dB	45 dB	−5.002*	1.877	0.019	–9.028	–0.976
	55 dB	7.093	5.248	0.198	–4.163	18.350
	65 dB	–4.019	3.651	0.290	–11.849	3.812
45 dB	55 dB	12.095*	4.994	0.030	1.384	22.807
	65 dB	0.983	3.620	0.790	–6.782	8.748
55 dB	65 dB	−11.112*	2.771	0.001	–17.055	–5.169

*Mauchly’s test of sphericity p = 0.001 < 0.05, which did not obey the hypothesis of spherical distribution, and the multivariate test result was p = 0.004 < 0.05. Based on estimated marginal means.*

*The mean difference was significant at the 0.05 level.

*^b^Adjustment for multiple comparisons: Least Significant Difference (equivalent to no adjustments).*

#### Low Frequency/High Frequency

The variation trend of LF/HF in the low-sensitive group (Figure 5) revealed that LF/HF increased at first and then decreased with the increase of SPL. The LF/HF increased obviously from 15.7 dB (A) to 45 dB (A), and the LF/HF values of 45 dB (A) and 55 dB (A) were similar, reaching a maximum offset to the baseline. The Mauchly’s test of sphericity of LF/HF (*p* = 0.010 < 0.05) did not obey the hypothesis of spherical distribution, and the further multivariate test was *p* = 0.412, which indicates that there was no significant difference in LF/HF between the different SPL levels.

#### Heart Rate

The variation trend of HR in the low-sensitive group (Figure 5) revealed a general decrease at first and then an increase with the increase of SPL, whereby HR decreased obviously from 15.7 dB (A) to 45 dB (A), and significantly fluctuated from 45 dB (A) to 65 dB (A). The maximum value of HR offset to the baseline appeared at 45 dB (A). The repeated-measures ANOVA result of HR for the main effect of SPL was *p* = 0.050, and the corresponding results of pairwise comparison (Table 6) revealed that HR was significantly different between 55 dB (A), 65 dB (A), and 45 dB (A) (all, *p* < 0.05).

**TABLE 6:** Low-sensitive group’s HR/bpm pairwise comparisons.

(I) SPL	(J) SPL	Mean difference (I-J)	Std. error	Sig.^b^	95% confidence interval for the difference^b^	
					Lower-bound	Lower-bound
15.7 dB (Resting)	35 dB	2.467	1.272	0.073	−0.261	−0.261
	45 dB	2.867	1.737	0.121	−0.859	−0.859
	55 dB	–1.733	2.892	0.559	−7.937	−7.937
	65 dB	0.467	2.065	0.824	−3.963	−3.963
35 dB	45 dB	0.400	1.154	0.734	−2.075	−2.075
	55 dB	–4.200	2.187	0.075	−8.891	−8.891
	65 dB	–2.000	1.298	0.146	−4.785	−4.785
45 dB	55 dB	−4.600*	2.042	0.041	−8.980	−8.980
	65 dB	−2.400*	1.018	0.033	−4.583	−4.583
55 dB	65 dB	2.200	1.455	0.153	−0.920	−0.920

*Mauchly’s test of sphericity p = 0.001 < 0.05, which did not obey the hypothesis of spherical distribution, and the multivariate test result was p = 0.050. Based on estimated marginal means.*

*The mean difference was significant at the 0.05 level.

*^b^Adjustment for multiple comparisons: Least Significant Difference (equivalent to no adjustments).*

## Analysis and Discussion

### Non-significant Difference Between Noise-Sensitive Groups

According to the results presented in section “The Differences in Significance of Heart Rate Variability Between Noise-Sensitive Groups,” the HRV indicators, including SDNN, LF/HF, and HR, did not differ significantly between the high-sensitive group and the low-sensitive group under short-term noise stimulation in this study. And these result are not consistent with the conclusions of previous relevant studies. In the chronic noise-physiological effect studies, cross-sectional studies of long-term exposure to traffic noise (mainly airport noise) (Babisch et al., 2009; Shepherd et al., 2010) confirmed that residents with higher noise sensitivity suffer significantly more CDs and sleep disorders than residents with lower noise sensitivity. And in the acute effect studies, Shepherd et al. (2016) confirmed that HRV of 103 subjects showed significant differences in SDNN and HR between different sensitive groups under stimulation of standard digitized sound samples [70 dB (A)] in laboratory. Compared with these studies, firstly, the level of noise in above studies were higher, while this study focuses on the effects of noise below 65 dB (A). Based on the physiological mechanism for coping with noise (Babisch, 2006; WHO Regional Office for Europe, 2018a), higher SPL stimulation may cause faster physiological feedback. Due to the low SPL [≤65 dB (A)] and short duration of noise stimulation (10 min) in this study, the slow physiological response caused by low SPL noise probably has not reached the adequate degree in a short time, so no significant results were presented. Secondly, compared with Shepherd’s study, the state of subjects and duration of noise stimulation during the experiment were similar in this study, but Shepherd chose digital audio samples which are different with the real recorded signals using in this study. And the HRV data testing period in Shepherd’s study was several seconds after noise exposure, while it was 10 min while noise stimulating in this study. In summary, the non-significance results in this study may be mainly caused by the lower SPL of stimuli noise followed by the characteristics of stimulating noise signals and HRV data testing period.

Although there were no significant differences in HRV of short-term noise stimulation between groups, this study still showed that there were clear differences in variation trend of the physiological indicators with SPL changing between the two sensitive groups. And according to WHO Regional Office for Europe (2018a) relevant theories, the acute biological effects of cardiovascular autonomic arousal do not adapt over time, and that long-term noise exposure can increase the risk of chronic diseases. Based on this theory, it could be inferred that non-significant differences between the two groups may cause distinction on physiology after long-term accumulating, and the details of HRV trends is worth further exploring.

### The Differences of Heart Rate Variability Trends Between Noise-Sensitive Groups

**FIGURE 6:**
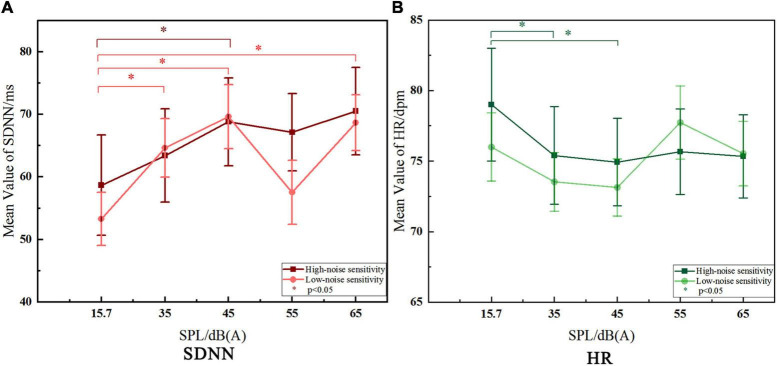
Comparison of the trends of SDNN and HR between noise-sensitive groups.

The results (Figure 6) show that there were significant variations just in SDNN and HR with the stimulating SPL changing in the both two groups, while LF/HF showed no significant change among different SPL. Therefore, only the characteristics of trends in SDNN and HR are discussed in this part.

#### Variation Trend of Standard Deviation of NN Intervals

Standard deviation of NN intervals showed an upward trend with the increase of SPL in both groups, which is consistent with relevant research conclusions (Kraus et al., 2013; Walker et al., 2016; El Aarbaoui et al., 2017). It follows that, in this study, under the influence of indoor-level road traffic noise, the higher SPL is, the higher SDNN value is. As we know, higher SPL has a greater impact on human body, confirmed by epidemiological studies on the chronic effects of noise, which shown that prevalence rate of CDs of residents living in high noise exposure environment for a long time was significantly higher than those living in quiet areas (Nassur et al., 2019). So it follows that higher SDNN value is detrimental to human physiological health. From this point of view, SDNN of the high-sensitive group was obviously higher than that of the low-sensitive group under 55 dB (A) and 65 dB (A) SPL, which the numerical difference was 9.595 ms and 1.868 ms, respectively. It can be seen that under the same noise level [55 dB (A) or 65 dB (A)], the high-sensitivity group is more affected.

On the other hand, some scholars suggest that rapid physiological responses of low-sensitive people were manifested as strong adaptability to noise (Stansfeld, 1992), and may give rise to the coping mechanism of humans mentioned by WHO which may indirectly reduce the impact of noise on health. In terms of the significance of changes in SDNN within groups, the high-sensitive group just showed a significant change to the baseline at 45 dB (A), while the low-sensitive group changed significantly almost with every increase of SPL level. It can be seen that the low-noise sensitive group is more responsive to the change of SPL. Meanwhile, in terms of the degree of deviation from the SDNN baseline, a similar status was observed. At 45 dB (A) (*p* < 0.05), there was a increase of 10.133 ms in the high-sensitive group and a increase of 16.343 ms in the low-sensitive group. It can be seen that under the same noise stimulus, the physiological feedback of the high-sensitive group was 6.21 ms slower, and the low-sensitive group also showed stronger response. Based on the analysis of the above two aspects, the low-sensitivity group showed a rapid response in SDNN than the high-sensitivity group. According to the above views of relevant scholars, the high-sensitivity group would be more affected by road traffic noise.

#### Variation Trend of Heart Rate

Heart rate showed a downward trend at lower SPL, then leveled off and even increased when SPL is higher than 45 dB (A) (only in the low-sensitive group showed an upward trend at higher SPL). According to Park and Lee (2017), under noise stimulation below 60 dB (A), HR decreases in the post-encounter stage due to people’s attention being oriented by stimulation (Lang and Bradley, 1997; Boucsein, 2012). After that, when the body reaches the high arousal noise stimulus state, HR presents an upward trend, corresponding to the circa-striker stage. From the above point of view, after significant decrease at 35 dB (A) and 45 dB (A) compared with the baseline, HR then leveled off in the high-sensitive group. However, the HR in the low-sensitive group increased significantly when SPL was higher than 45 dB (A), reaching earlier to the upward trend than the high-sensitive group, which is correspond to the circa-striker stage mentioned above. As other studies have shown, HR of the high-sensitive group seemed to be less responsive to different noise stimuli than that of the low-sensitive group (White et al., 2010), and the ability to adapt to noise was also weaker (Stansfeld, 1992). And this is consistent with the result reflected by SDNN.

In terms of HR value, an epidemiological study of people living with long-term exposure to aircraft noise in west London found that HR value of high-sensitive groups was lower than that of low-sensitive groups (Stansfeld et al., 1985). This study also found a similar trend under the short-term influence of noise, which was lower 2.1 dpm at 55 dB (A) and 0.2 dpm at 65 dB (A) in HR of the high-sensitive group than that of the low-sensitive group. At the same time, the above-mentioned 55 dB (A) and 65 dB (A) (also in the SDNN part) are highly consistent with the SPL findings of epidemiological studies that the prevalence of CDs increasing significantly when the SPL is higher than around 55 dB (A) to 60 dB (A) (Barregard et al., 2009; Bluhm and Eriksson, 2011; Chang et al., 2011). And according to the WHO cumulative effect of noise impact, the differences in HR (and in SDNN) indicators observed in this study caused by short-term noise stimulation may have the potential to produce physiological response and lead to between-groups differences in prevalence after long-term recurrent effect, which is worthy of further research.

## Conclusion and Prospects

In this study, there was no significant difference between sensitive groups in the acute physiological effects of indoor-level traffic noise reflected by observing SDNN, LF/HF, and HR. According to the comparative analysis with the previous research, the possible reasons may be related to the low SPL [≤65 dB (A)] of noise concerned in this study, as well as the type of stimulus audio signal and HRV data testing period.

However, according to the theory of cumulative effect of noise proposed by WHO Regional Office for Europe (2018a), we discussed the variation trends and the within-group significant changes of SDNN and HR indicators with SPL, and the results showed that the high-sensitivity group was more affected by road traffic noise. In addition, there are also observable numerical differences in SDNN and HR between the two sensitive groups at the SPL that can significantly increase the prevalence of CDs concerned by epidemiological studies. According to the cumulative effect theory, these non-significant but observable differences in HRV are likely to lead to differences in morbidity between groups, and deserve attention and further research.

In the follow-up study, it is necessary to focus on the residents living on the street for a long time, and the relationship between the acute and chronic noise effects needs to be further discussed by observing the differences between exposed groups and non-exposed groups. For another, the subjects of this study were only college students. Because of the prevalence of CDs would increase with age, middle-aged and elderly population should be paid attention to in subsequent studies. In addition, in order to further improve the experiment, the acquisition and playing process of noise signals should be optimized.

## Data Availability Statement

The original contributions presented in the study are included in the article/supplementary material, further inquiries can be directed to the corresponding author/s.

## Ethics Statement

The studies involving human participants were reviewed and approved by the School of Architecture, Tianjin Chengjian University, Tianjin, China. The patients/participants provided their written informed consent to participate in this study. Written informed consent was obtained from the individual(s) for the publication of any potentially identifiable images or data included in this article.

## Author Contributions

CC and YW conceptualized and supervised the project. YNX managed the project and did data analysis and graphs. CC, YNX, and QKW designed the experiments. YNX and QKW carried out the experiments. CC and YNX conceptualized and wrote the first draft of the manuscript. LL made substantial contributions in searching for references. All authors participated in reviewing the manuscript.

## Conflict of Interest

The authors declare that the research was conducted in the absence of any commercial or financial relationships that could be construed as a potential conflict of interest.

## Publisher’s Note

All claims expressed in this article are solely those of the authors and do not necessarily represent those of their affiliated organizations, or those of the publisher, the editors and the reviewers. Any product that may be evaluated in this article, or claim that may be made by its manufacturer, is not guaranteed or endorsed by the publisher.

## Footnotes

1 Only *L*_d_ is available in the China Environmental Noise Prevention and Control Annual Report.

2 The physiological data measured at resting state (under 15.7 dB) were taken as the physiological baseline.
